# Sex‐Specific Differences in the Secretome of Oligodendrocyte Progenitor Cells Post Hyperoxic Stress

**DOI:** 10.1002/jex2.70082

**Published:** 2025-09-23

**Authors:** Donna Elizabeth Sunny, Elke Hammer, Stephan Michalik, Uwe Völker, Matthias Heckmann

**Affiliations:** ^1^ Department of Neonatology and Pediatric Intensive Care University of Medicine Greifswald Greifswald Germany; ^2^ Department of Functional Genomics University of Medicine Greifswald Greifswald Germany; ^3^ DZKJ (German Centre for Child and Adolescent Health) Partner Site Greifswald/Rostock Greifswald Germany

**Keywords:** hyperoxia, murine oligodendrocyte progenitor cells, sectretome, sex‐differences

## Abstract

Cerebral oxygenation differences in the neonatal period of human preterm infants, along with sex‐specific differences in combating oxidative stress, can lead to disruption of normal oligodendrocyte maturation and function, which in turn can differentially affect neuronal development and activity in the male and female brains. Secretory proteins and extracellular vesicles (EVs) are increasingly recognized as important mediators of intercellular communication and stress response in the brain. Our analysis of the secretome from cell culture supernatants obtained after treating male and female derived primary mouse OPCs with hyperoxia (80% O_2_) for a 24 h period showed prominent sex‐specific protein signatures with only 6% intersection between sexes upon hyperoxia. A higher proportion of mitochondrial proteins was observed to be secreted by male cells upon hyperoxic stress. Among specific factors that could be identified exclusively in the hyperoxia‐treated groups, FGF‐2 was present in significantly higher amounts in the female supernatant. Functional assays on neuronal cells (male) revealed that treatment with supernatant from female hyperoxic OPCs resulted in increased neuronal viability, potentially due to elevated levels of FGF‐2. This suggests that female‐specific extracellular proteins may play a key role in sex specific stress response and are potential candidates for further investigation.

## 1 Introduction

Effective communication between the different cell types in the central nervous system (CNS) is important for proper neural development, maintenance and regeneration (Fruhbeis et al. 2020). At the same time, cell‐cell communication might also play an important role under pathological and stress conditions (Fruhbeis et al. 2013). This intercellular communication is achieved by secretory factors that are taken up by the neighbouring cells, where they either trigger specific signalling pathways and cellular responses or are utilized by the cells for the maintenance of the cellular machinery. These secretory factors may contain freely secreted proteins as well as proteins that are enclosed in extracellular vesicles (EVs).

During brain development, oligodendrocytes play a key role in generating large amounts of myelin membrane that wraps around axons, providing trophic support and the necessary electric insulation for neuronal signal transduction. EVs are known to be released by mature oligodendrocytes (OLs) and, owing to their intimate interaction with neurons, they are known to be taken up by neurons in response to different signalling and even exhibit neuroprotective properties (Kramer‐Albers 2020). However, not much is known about EV secretion by the oligodendrocyte progenitor cells (OPCs) during development. Moreover, EV secretion could also be associated with the secretion of soluble proteins that could also play an important role in signalling, especially under stress conditions. These secretory proteins may execute a broad spectrum of functions, which are not yet completely understood.

Especially during the early developmental phases of the brain where the progenitor population dominates, any insult involving oxidative stress which is common due to the use of oxygen in the neonatal intensive care treatment of preterm infants, has a tremendous impact on the developing brain and is a major factor contributing to white matter damage in the preterm brain (Reich et al. 2016; van Tilborg et al. 2018; van Westering‐Kroon et al. 2021). This is associated with a clear distinction in the outcome between both sexes (Lauterbach et al. 2001; Morsing et al. 2011). Male sex has been identified as a major risk factor for poor neurological outcome across multiple studies in the most vulnerable patient population of preterm infants (Barnett et al. 2018; Hintz et al. 2006). Unfortunately, we still lack a clear view of the molecular mechanisms that lead to such profound sex‐based differences.

In our previous work on primary murine OPCs, we have shown that prominent sex based differences in the response of OPCs towards oxidative stress exist (Sunny et al. 2020). This led to the hypothesis that these sex‐based differences might also be present in the extracellular signalling generated by these cells in response to oxidative stress.

In the current study, we characterized the differences between the secretomes of male and female‐derived murine OPCs. We demonstrate that the overall secretome profiles of male and female cells differ with very little overlap under normal conditions, as well as post‐hyperoxic treatment. This suggests the possible activation of differential pathways in male and female cells in response to oxidative stress that leads to the generation of differential extracellular signals. Moreover, we show that hyperoxic stress can trigger the secretion of specific growth factors, such as FGF‐2, in a sex‐specific manner. These factors exhibit neurotrophic activity by differentially affecting the survival and viability of cultured neuronal cells.

## 2 Results

### 2.1 Comparison of Protein Profiles of Male and Female Murine OPCs

In our model system, murine OPCs were plated for a duration of 24 h, under normoxic (3% O_2_) and hyperoxic conditions (80% O_2_), respectively. Thereafter, we isolated the total secreted proteins from male and female‐derived OPC supernatants. In the samples, we measured intensities of 3268 proteins. An initial analysis to identify the effect of sex as the main variable was performed by looking at the total number of significantly differentially abundant proteins, where comparisons were done between male and female under normoxia and hyperoxia separately (M vs. F_N and M vs. F_H). For the majority of detected proteins, no significant differences in levels were observed in both male and female supernatants. The number of proteins with significantly different intensities in the comparison groups was rather small (3%) in relation to the total number of identified proteins, and this was similar under normoxic (105 proteins) and hyperoxic (104 proteins) conditions.

**FIGURE 1:**
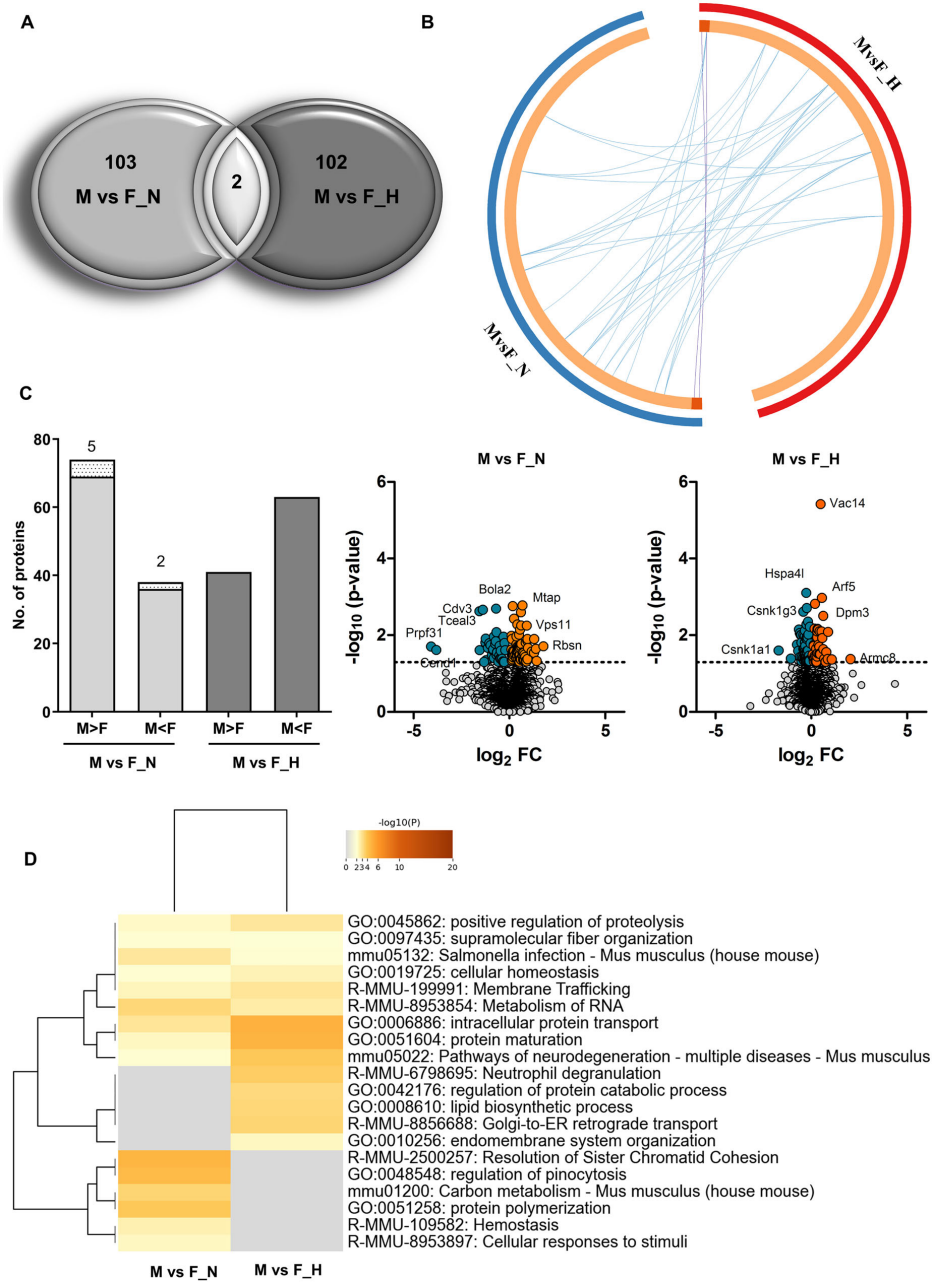
Sex‐dependent differences in the secretome of OPCs. (A) Venn diagram of differentially abundant proteins in male (M) and female (F) samples under normoxic conditions (M vs. F_N) and post 24 h 80% O_2_ hyperoxia treatment (M vs. F_H). Cut off *p* value being 0.05. (B) Circos plot showing overlap of differentially abundant proteins in male and female groups at normoxic and hyperoxic conditions. Cut off *p* value being 0.05. Dark orange colour represents the proteins that are shared by the two groups, and light orange colour represents proteins that are unique to the groups. Purple lines link the proteins that are shared by both groups. Blue lines link proteins, which belong to the same structural, functional or gene ontology terms and which are significantly enriched in these terms. (C) *Left panel*: Bar graph showing numbers of proteins with different levels in male and female samples under normoxic (light grey) and hyperoxic (dark grey) conditions. Sex chromosome‐linked proteins are shown as stacked bars, and numbers are shown on top (only X‐linked proteins appear as only one Y‐linked protein was identified in the dataset, which was not statistically significant in any of the comparison groups). *Right panel*: Volcano plots representing differential abundance of proteins in the M versus F_N and M versus F_H groups. Mapped log_2_‐ratios are depicted with a colour scale (M < F blue, M > F red). The cutoff *p* value being 0.05. (D) Metascape analysis dendrogram showing the statistically enriched terms (e.g., GO/KEGG terms, canonical pathways, hallmark gene sets), revealed from proteins differentially abundant between male and female samples at normoxic and hyperoxic conditions. The heatmap cells are coloured by their *p* values, white cells indicate the lack of enrichment for that term in the corresponding protein list. Cut off *p* value being 0.05. All data are derived by the analysis of three independent experiments.

Surprisingly, very little overlap was observed between the proteins identified in both comparisons (Figure 1A), as well as little functional overlap between the different proteins as determined by metascape analysis (Zhou et al. 2019) (Figure 1B). Further, a greater number of proteins showed higher abundance in the male secretome as compared to the female secretome under normoxic conditions. However, this trend reversed upon hyperoxia, where a greater number of proteins were less abundant in the male secretome as compared to the female secretome (Figure 1C). Further functional analysis using Metascape showed enrichment of intracellular protein transport and pathways of neurodegeneration and multiple diseases in the differentially abundant proteins upon hyperoxia (Figure 1D). The evidence presented here indicates the presence of intrinsic sex‐specific differences in the secretome of male and female cells, which are visible under hyperoxic as well as normoxic conditions. Therefore, we conducted an analysis of the contribution of sex‐chromosome‐coded genes to the observed differences. Under normoxic conditions, 6.6% of the significantly different proteins (*p* value < 0.05) were linked to sex chromosomes, whereas none of the sex‐linked proteins were significantly different between males and females post hyperoxia (Table S1). Thus, hyperoxic treatment diminishes X‐linked differential protein levels, but at the same time, other sex‐specific alterations occur. Overall, the observed differences remained largely non‐overlapping.

### 2.2 Hyperoxia Alters the Secretome Profile of Male and Female OPCs

**FIGURE 2:**
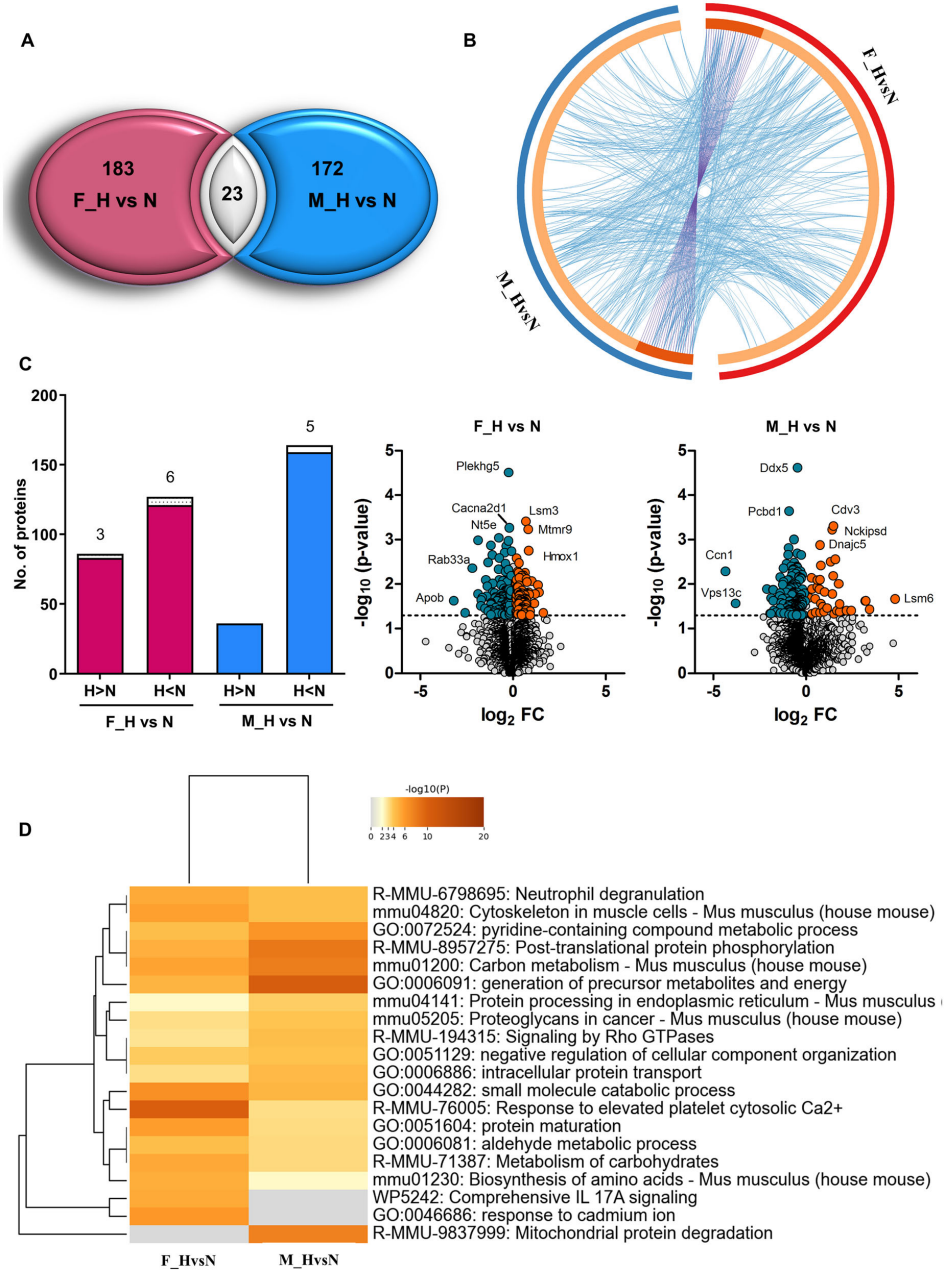
Effect of hyperoxia on the secretome of male and female OPCs. (A) Venn diagram of proteins displaying significantly different abundance between normoxic and hyperoxic conditions in culture supernatants of male (M_H vs. N) and female (F_H vs. N) murine OPCs, respectively (*p* value < 0.05). (B) Circos plot showing overlap of proteins displaying significantly different abundance between normoxic and hyperoxic conditions in culture supernatants of male (M_H vs. N) and female (F_H vs. N) murine OPCs, respectively (*p* value < 0.05). Dark orange colour represents the genes that are shared by the two groups (M_H vs. N and F_H vs. N), and light orange colour represents genes that are unique to the groups. Purple lines link the proteins that are shared by both groups. Blue lines link the proteins, which belong to the same structural, functional or gene ontology terms and which are significantly enriched in these terms. (C) *Left panel*: Bar graph showing numbers of proteins with altered levels in male (blue) and female (red) samples upon hyperoxic conditions compared to normoxic control conditions. Sex chromosome‐linked proteins are shown as stacked bar, and numbers are shown on top (only X‐linked appear as only one Y‐linked protein was identified). *Right panel*: Volcano plots showing all significantly altered proteins in the M_H versus N and F_H versus N groups. Mapped log_2_‐ratios are depicted with a colour scale (M < F green‐blue, M > F orange). The cut off *p* value being 0.05. (D) Metascape analysis dendrogram showing the statistically enriched terms (GO/KEGG terms, canonical pathways, hallmark gene sets, etc.), based on significantly altered proteins upon hyperoxia treatment (F_H vs. N and M_H vs. N). The heatmap cells are coloured by their *p* values, white cells indicate the lack of enrichment for that term in the corresponding protein list. Cut off *p* value being 0.05. All data are derived by the analysis of three independent experiments.

In order to further assess the effect of hyperoxia on the secretome profile, we looked at the sex‐specific response to hyperoxic conditions (M_H vs. N and F_H vs. N). The effect of the high oxygen concentration on the secretome was stronger (male 5.9% and female 6.3%) than the sex‐ specific differences (3%). Even though the significantly altered proteins in males and females showed very little overlap (Figure 2A), a quite high functional overlap was observed when considering multiple ontology terms, including structural, functional and gene ontology (Figure 2B). Next, we used Metascape functional analysis to identify the response‐related pathways and biological processes.

This revealed an enrichment of functions like small molecule catabolic process and protein maturation in the female group upon hyperoxia, whereas, in the male group, altered proteins were enriched in post‐translational protein phosphorylation, generation of precursor metabolites and energy, and mitochondrial protein degradation (Figure 2D). The overall effect of hyperoxia was a decreased level of a high number of proteins in the secretome of cells of both sexes (76.5% of all significantly altered proteins in the case of males and 57.9% in the case of females). However, a larger portion of proteins increased in abundance upon hyperoxia in the female secretome (42%) as compared to the male secretome (23.4%). However, the effects were stronger in the male secretome as observed by the ratio values and shown in the volcano plots. Moreover, a slightly higher number of X‐chromosome‐linked proteins were seen to be significantly regulated in the female secretome in response to hyperoxia (Figure 2C).

Despite the limited number of biological replicates (*n* = 3), we performed a two‐factor ANOVA analysis to assess the interaction between sex and oxygen treatment, and the resulting significance values are included in Table S1.

**FIGURE 3:**
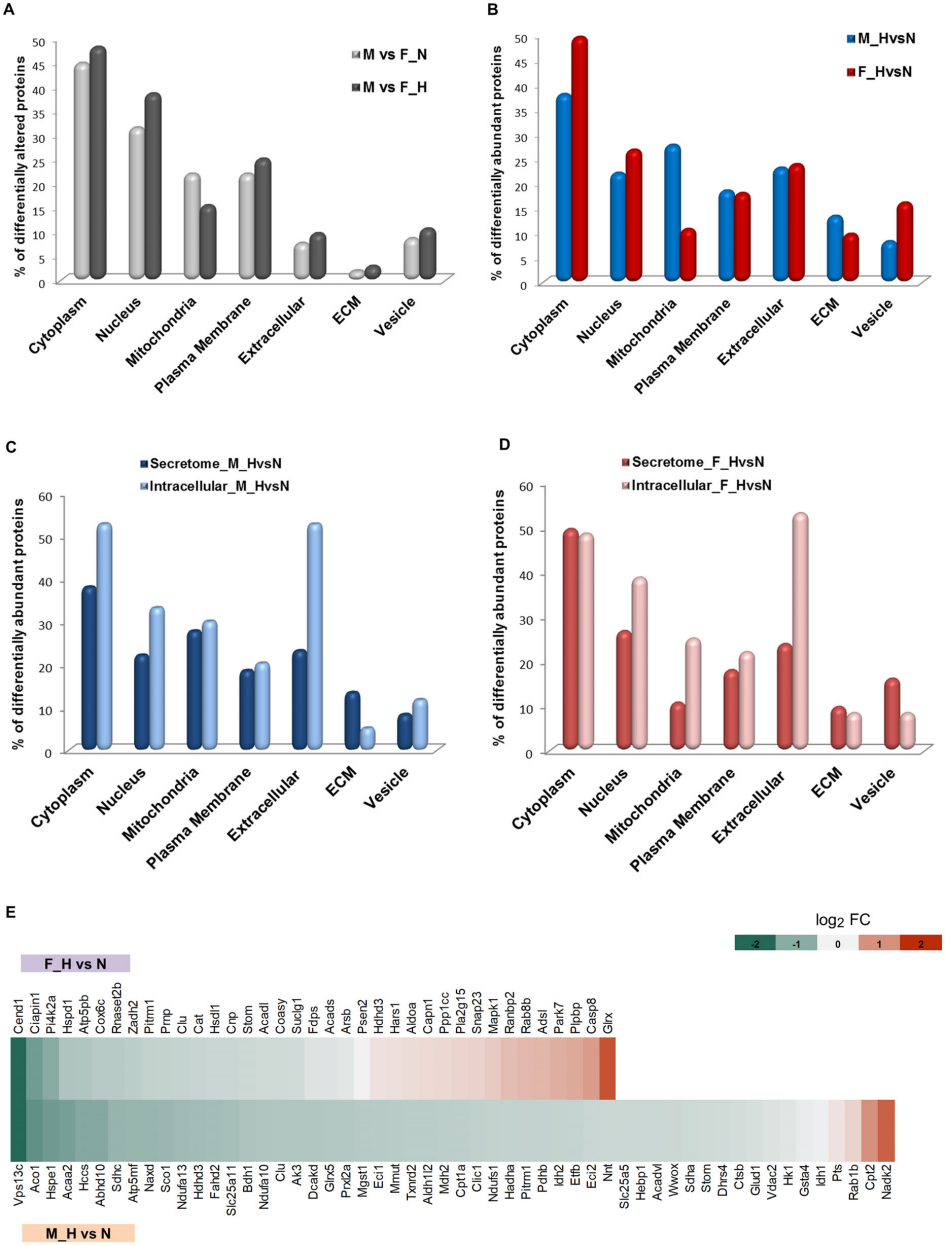
Effect of hyperoxia on the secretome and intracellular proteome of male and female OPCs. Bar charts showing the percentage of proteins displaying significantly different sex‐specific abundance under normoxic or hyperoxic conditions (M vs. F) or after the impact of hyperoxic conditions (H vs. N) in samples from male and female OPCs. Proteins are grouped according to their gene ontology cellular component data (UniProt) by the terms indicated in the x‐axis. (A) Protein abundance differences in supernatants of male and female samples under normoxic conditions (light grey) and hyperoxic conditions (dark grey). (B) Protein abundance differences in supernatants between hyperoxia compared to normoxia in male cells (blue) and female cells (dark red). (C, D) Comparison of hyperoxia effects in the secretome (dark blue and red) and intracellular proteome (light blue and red) for male (C) and female (D) OPC samples, respectively. (E) Heatmap representation of the log_2_ fold‐change (FC) ratios of all mitochondrial proteins displaying significantly altered abundance in the M_H versus N and F_H versus N groups. Mapped log_2_‐ratios are depicted with a colour scale. Proteins are represented by their more commonly used gene names.

Next, we focused on the different fractions of proteins secreted by the male and female OPCs under normoxic and hyperoxic conditions, based on their gene ontology‐based cellular component by UNIPROT. Proteins were sorted according to their assigned cellular localization, and the numbers were counted for each specific group analyzed. Comparing the sex differences at normoxic and hyperoxic conditions, a higher number of mitochondrial proteins (normoxic: 22%; hyperoxic: 15%) showed sex‐specific levels under normal conditions, as compared to hyperoxic conditions (Figure 3A).

Proteins were quantified and counted based on at least three biological replicates. Proteins can have multiple UniProt annotations, for example, for plasma membrane, cytoplasm, nucleus, and so forth, and are thus grouped to more than one category. Cut off *p* value being 0.05.

But looking at the hyperoxia effect in the male and female groups separately, we saw approximately 17% more mitochondrial proteins being differentially regulated in the male secretome, along with approximately 4% more extracellular matrix (ECM) proteins. In the female cells, higher numbers of vesicles (∼8%) and cytoplasmic proteins (∼12%) were differentially regulated (Figure 3B). The higher proportion of vesicle proteins in the female group could indicate increased release of membrane‐bound vesicles from female cells upon hyperoxic stress. In order to better understand the secretory pattern of these cells, we further analyzed the secretome in comparison to the intracellular proteome (Sunny et al. 2020). The comparisons showed an overall similar pattern in the male and female cells. But it was interesting to see that the fraction of mitochondrial proteins that were altered in the secretome of male cells post hyperoxia was almost similar to the percentage of significantly altered mitochondrial protein fraction in the cells intracellularly (Figure 3C,D). This points to a certain susceptibility of mitochondria‐associated proteins to hyperoxic stress in the male cells. Therefore, we took a closer look at the regulation of these detected mitochondrial proteins in the male secretome. Upon hyperoxic stress, only a few proteins were strongly upregulated, which included endophilin‐B1 (Sh3glb1), carnitine O‐palmitoyltransferase 2 (Cpt2) and mitochondrial NAD kinase 2 (Nadk2). In contrast, the majority of the proteins showed a significant decrease in abundance post‐hyperoxia (Figure 3E). Endophilin‐B1, also known as Bax‐interacting factor 1 (Bif‐1), is a multifunctional protein that regulates apoptosis (Takahashi et al. 2007), autophagic cell death and mitochondrial function (Takahashi et al. 2005), which is also implicated in brain pathologies like Alzheimer's disease (Wang et al. 2015). It was interesting to note that the intracellular Bif‐1 levels in male OPCs showed a slight decrease post‐hyperoxic treatment as observed from the proteomic data (data not shown). This might support an increased secretion of Bif‐1, leading to lower intracellular levels which could result in the eventual negative outcome on the survival of male OPCs post hyperoxia. Moreover, a recent study demonstrated a positive correlation of glutaredoxin‐1 (Glrx) protein with favourable neurodevelopmental outcomes in preterm infants (Zhao et al. 2024). As a strong upregulation of Glrx protein was observed in the female secretome post hyperoxia, this might as well aid in providing a better antioxidant defence mechanism in the female brain.

To rule out the possibility of cell death and lysis as contributing factors to the observed mitochondrial proteins in the secretome, we performed a series of complementary assays, including cleaved caspase‐3 immunostaining followed by flow cytometry, Western blot using active caspase 3 antibody, phase contrast microscopy, LDH release and XTT assays. The results as shown in Figure S1, indicated no signs of cell death, extensive apoptosis or cytotoxicity after 24 h of hyperoxia treatment. Though XTT assay revealed a significant decrease in metabolic activity in both male and female cells following hyperoxia (Figure S1E), indicating metabolic stress but not necessarily cell death. Finally, the secretome analysis also did not show intracellular proteins in intensities correlating to that of the analyzed cell lysates, which would be expected in the event of substantial cell lysis.

### 2.3 ON/OFF Targets of Male and Female Secretomes Differ Extensively Post Hyperoxic Treatment

We further looked at the proteins that were present only either under normoxic condition or post hyperoxia (ON/OFF targets) in both male and female groups separately (full list in Table S2). We identified brain‐specific serine protease 4 (BSSP‐4) (Prss22) to be present only under normoxic conditions in both male and female secretome. In contrast, neurosecretory protein VGF (Vgf) and plasmolipin (plasma membrane proteolipid) (Pllp) were identified exclusively in the female secretome under normoxic conditions. However, hyperoxia triggered the secretion of distinct proteins in male and female cells. Female cells secreted fibroblast growth factor 2 (FGF‐2), which is an important protein involved in a vast number of molecular pathways and a number of cellular functions including glial cell differentiation (Reuss et al. 2003) and response to axon injury (Jungnickel et al. 2005; Timmer et al. 2007). Other proteins included vacuolar protein sorting‐associated protein 37C (Vps37c), aryl hydrocarbon receptor nuclear translocator 2 (Arnt2), the extracellular histone H1.1 (H1a) and bisphosphoglycerate mutase (Bpgm) (Table 1). Hence, female OPCs appear to exhibit a proactive protective response to oxidative stress, characterized by neurotrophic support (FGF‐2, VGF), efficient vesicular trafficking (Vps37c, H1a), metabolic adaptation (Bpgm) and stress signaling regulation (Arnt2). Moreover, the baseline secretion of Vgf and Pllp under normoxia suggests that female OPCs maintain a homeostatic advantage even before stress exposure, potentially contributing to greater resilience compared to males.

**TABLE 1:** Secretome ON/OFF targets.

Gene name	ON/OFF Female	Function	Reference
*Prss22*	N	Tumour cell migration and invasion	Chen et al. (2014)
*Vgf*	N	Roles in neurogenesis and neuroplasticity associated with learning, memory, depression and chronic pain	Jiang et al. (2019) Lin et al. (2015)
*Pllp*	N	Involved in myelination, intracellular transport and lipid raft formation	Azzaz et al. (2023), Shulgin et al. (2021)
Bpgm	H	Plays a major role in regulating haemoglobin oxygen affinity Known to increase oxyhaemoglobin and reduce energy metabolism under stress conditions	Qiang et al. (2021), Xu et al. (2020)
*Vps37c*	H	Component of the endosomal sorting complex required for transport (ESCRT)‐I Endosome transport via the multivesicular body sorting pathway and protein targeting to the membrane A regulator of vesicular trafficking process Cellular stress responses when they are associated with ESCRT‐I destabilization	Eastman et al. (2005), Kolmus et al. (2021)
*H1‐1*	H	Bound to vesicles Plays a role in the positive regulation of receptor‐mediated endocytosis	Brix et al. (1998)
*Arnt2*	H	Transcription factor Development of the hypothalamo‐pituitary axis and postnatal brain growth	Aitola and Pelto‐Huikko (2003), Hosoya et al. (2001)
*Fgf2*	H	Glial cell differentiation Response to axonal injury	Jungnickel et al. (2005), Reuss et al. (2003), Timmer et al. (2007)
	ON/OFF Male		
*Prss22*	N	Tumour cell migration and invasion	Chen et al. (2014)
*Rassf2*	H	Involved in epidermal growth factor receptor signalling pathway via I‐kappaB kinase/NF‐kappaB cascade Positive regulation of the apoptotic process and JNK cascade	Song et al. (2012) Song et al. (2010)
*Gnai1*	H	Involved in G protein‐coupled receptor signalling pathway Associated to paediatric encephalopathy	Solis et al. (2021)
*Arih2*	H	Plays an essential role in protein ubiquitylation and degradation	Martinez‐Noel et al. (1999)
*Pqbp1*	H	Associated with neurodegenerative disorders and brain inflammation	Jin et al. (2021)
*Mink1*	H	Regulator of dual leucine zipper kinase (DLK)/c‐Jun‐N‐terminal kinase (JNK) pathway signalling in neurons that drives both neurodegeneration and axon regeneration	Larhammar et al. (2017)

*Note*: Table representing selected proteins (depicted by their gene names) that were identified only in either the normoxic conditions (N) or hyperoxic conditions (H) in male and female samples, respectively, along with their reported functions. The column ‘ON/OFF Female’ represents proteins exclusively detected under normoxic or hyperoxic conditions in samples from female OPCs, and similarly ‘ON/OFF Male’ represents proteins in samples from male OPCs.

In case of the male samples, we identified Ras association domain‐containing protein 2 (Rassf2), guanine nucleotide‐binding protein G(i) subunit alpha‐1 (Gnai1) as well as E3 ubiquitin‐protein ligase ARIH2, and polyglutamine‐binding protein 1 (Pqbp1) which is known to be associated with neurodegenerative disorders and brain inflammation (Jin et al. 2021). Furthermore, misshapen‐like kinase 1 (Mink1) also associated with neurodegeneration (Larhammar et al. 2017), was present only in the supernatant of male cells upon hyperoxia (Table 1). The presence of proteins linked to paediatric encephalopathy, neurodegeneration and inflammation suggests that male OPCs might be more vulnerable to oxidative stress. It also indicates a hyperoxia‐induced shift towards pro‐apoptotic, inflammatory and neurodegenerative pathways.

### 2.4 FGF‐2 in the Female Hyperoxia Treated OPC Derived Supernatant Increases Viability of Neuronal Cells

Even though FGF‐2 was the fourth‐ranking protein in abundance in our proteomic data comparison of the ON/OFF targets in the female OPC secretome (Table S2), it was the only protein with a specific annotation for ‘extracellular space’ and hence the most promising candidate from which paracrine effects could be assumed. Therefore, we focused our further experiments on FGF‐2.

**FIGURE 4:**
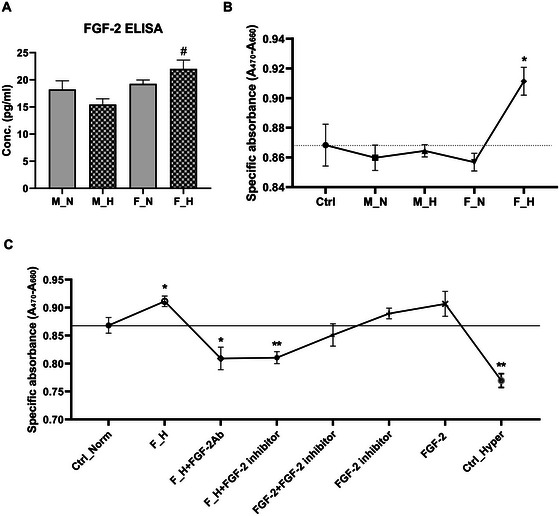
Functional analysis of the impact of FGF‐2. (A) FGF‐2 ELISA results showing the concentration of FGF‐2 in pg/mL as determined for each OPC supernatant sample type (N for 3% O_2_ normoxia; H: 80% O_2_ hyperoxia; M: male and F: female). (B, C) Graphs showing the effect of the different cell culture supernatants (B) and an FGF‐2 inhibitor (C) on the viability of differentiated ReNcell neurons under normoxic conditions as determined by XTT assay. ‘Ctrl’ refers to untreated ReNcell neurons in (B), Ctrl_Norm in (C) refers to untreated cells at normoxic conditions, Ctrl_Hyper refers to untreated cells kept under hyperoxic conditions for 24 h and FGF‐2Ab: treatment with FGF‐2 antibody. Increased specific absorbance values indicate higher cell viability. Data represent at least three biological replicates. # *p* < 0.05 compared to treatment with supernatant of male cells grown under hyperoxic conditions; * *p* < 0.05 or ** *p* < 0.01 compared to untreated ReNcells (Ctrl‐Norm).

Since secreted FGF‐2 could be a major factor that might influence the survival of the neighbouring cells under stress conditions, we performed an ELISA to determine the absolute amounts of FGF‐2 in the supernatant. The results showed a significantly higher amount of FGF‐2 in the supernatant of female cell culture upon hyperoxia as compared to male hyperoxia treated supernatant (Figure 4A). To further explore the functional effects of the secreted FGF‐2, we treated differentiated ReNcell neurons with the cell culture supernatants derived from male and female OPCs that were either treated with 80% O_2_ or were kept at normoxic conditions for 24 h. We subsequently performed an XTT assay to determine if the supernatants influenced the viability of the neuronal cells. The results demonstrate a substantial enhancement of neuronal viability when the neurons were exposed to female hyperoxia medium (Figure 4B). To determine if the observed enhancement in viability could be attributed to FGF‐2, XTT assays were performed where the neuronal cells were treated with female hyperoxia supernatant in combination with an FGF‐2 antibody and an FGF‐2 inhibitor, respectively. As shown in Figure 4C, treatment with either FGF‐2 antibody or FGF‐2 inhibitor significantly reduced the viability of the neuronal cells, which could be restored upon addition of FGF‐2‐containing medium.

### 2.5 Treatment With Female Hyperoxia Treated Supernatant Leads to Comparable Molecular Regulations in Neurons as by Addition of Pure FGF‐2

**FIGURE 5:**
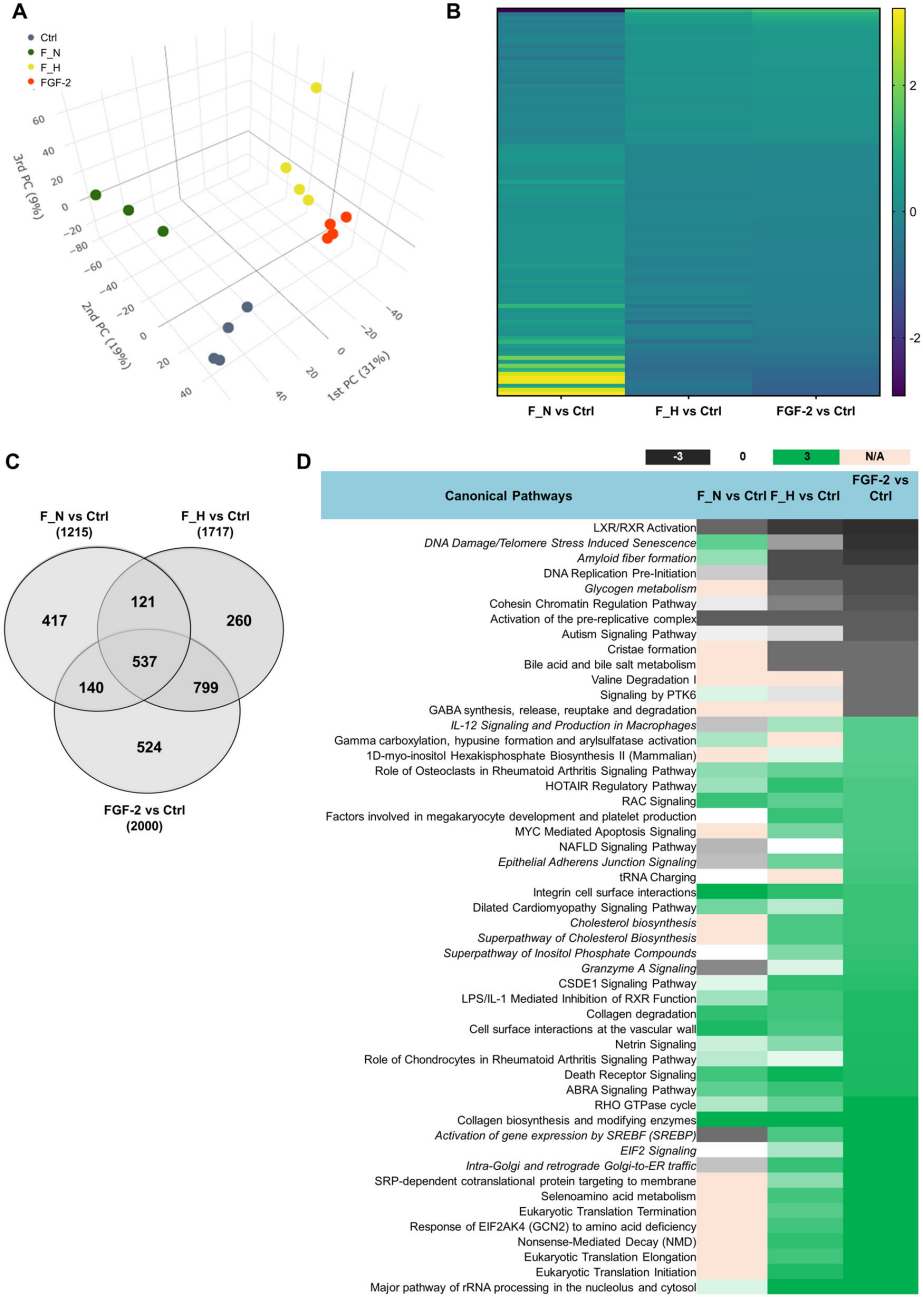
Effect of FGF‐2 treatment on ReNcell neurons. (A) Principal Component Analysis (PCA) plot showing the relationship of protein profiles of individual bio‐replicates of the untreated Ctrl (green), and treated samples (F_N, blue; F_H, yellow and FGF‐2, orange). The analysis yielded four distinct clusters, which were found to be correlated with the respective treatment of the samples. The greatest similarity was observed for the F_H and FGF‐2‐treated samples. (B) Heat‐map representation of all significantly altered proteins that were oppositely regulated in the F_N supernatant treated versus untreated Ctrl (F_N vs. Ctrl), in comparison to F_H supernatant treated versus untreated Ctrl (F_H vs. Ctrl), and FGF‐2 versus untreated Ctrl (FGF‐2 vs. Ctrl) groups. Log_2_‐ratios are depicted with a colour scale as shown in the figure. A cutoff *p* value of 0.05 was applied. (C) The Venn diagram illustrates the comparison of proteins displaying significantly altered abundance between F_N versus Ctrl, F_H versus Ctrl and FGF‐2 versus Ctrl groups. An adjusted cut‐off *p* value of 0.05 was used. (D) Heat‐map representation of the *z*‐score values from canonical pathways identified by analysis with IPA software due to enrichment of proteins displaying altered abundance upon FGF‐2 treatment in comparison to untreated female cells (*z* > |2|). Results are shown in comparison with F_N versus Ctrl and F_H versus Ctrl groups. *Z*‐score values are depicted with a colour scale, with green indicating activation and black indicating inhibition. The pathways for which an activation pattern could not be predicted are represented in pale orange colour. A cutoff of 0.05 adjusted *p* value and 1.2‐fold change was used for analysis. All data are representative of at least three independent experiments.

Further, a comparative proteomic analysis was performed on differentiated ReNcell neurons that were either used untreated as a reference sample or treated with female hyperoxia or normoxia supernatants or medium containing pure FGF‐2. The objective of this analysis was to see whether comparable proteomic changes and molecular regulations could be triggered. The measurement of the samples in data‐independent mode allowed the quantification of >6000 proteins, enabling a comprehensive analysis of the treatment effects on the intracellular proteome of the human ReNcells. A complete list of identified proteins is provided as Table S3. Principal component 1 revealed a clustering of the control samples together with the samples treated with supernatants from female normoxic samples on one side and the groups treated with FGF‐2 only and female hyperoxia supernatants on the other (Figure 5A). The majority of the proteins in the hyperoxia supernatant‐treated group were regulated in the same direction as by FGF‐2, as shown in the heatmap (Figure 5B). The overall significant protein changes showed a 70% overlap with those observed after treatment with hyperoxia supernatant, but only a 40% overlap when cells were incubated in the presence of normoxia supernatant (Figure 5C). Canonical pathway analysis using IPA revealed more similar pathways between groups treated with FGF‐2 and hyperoxia supernatant than those incubated with normoxia supernatant (Figure 5D). Looking at the pathway regulations, we observed an activation of *Epithelial Adherens Junction Signaling*, *Granzyme A Signaling* and *Activation of gene expression by SREBF (SREBP)* in the FGF‐2 and hyperoxia groups, which were inactivated or not regulated in the normoxic group. On the other hand, *DNA Damage/Telomere Stress Induced Senescence* and *Amyloid Fiber Formation* were seen to be activated in the normoxia group and inactive in the other two groups (Figure 5D).

We observed increased intracellular FGF‐2 levels in all three groups, but decreased FGFR. This might be due to ligand‐induced downregulation as a result of binding of extracellular and intracellular FGF‐2 to FGFRs, leading to receptor internalization and subsequent degradation to prevent overactivation of the signalling pathway, a mechanism similar to epidermal growth factor receptors (Ornitz and Itoh 2022; Soubeyran et al. 2002). Moreover, we see an increase in two of the Sprouty proteins (SPRY2 and SPRED1), which can modulate the activity of FGFRs and downstream signalling pathways (Guy et al. 2003; Yusoff et al. 2002).

## 3 Discussion

Hyperoxia is well known to hinder the maturation of OPCs, being a worrying cause of white matter damage in the preterm brain (Gerstner et al. 2008; Saugstad 2001). Considering accumulating evidence from clinical studies mentioning male sex as an independent associated risk factor (Hintz et al. 2006; Ingemarsson 2003; Mayoral et al. 2009; Sutton and Darmstadt 2013), it became apparent that the male and female brains could respond differently to hyperoxic insult.

Extending our previous studies (Sunny et al. 2021, 2020, 2022), which focused on intracellular signalling, we now reveal distinct differences between the secretomes of male and female‐derived OPCs, both under normal conditions and in response to high oxygen. These differences suggest that male and female OPCs might communicate differently with their environment and neighbouring cells, and that male and female OPCs might have different mechanisms for coping with or mitigating oxidative damage.

Looking at the sex‐specific secretory protein patterns (Figure 3), it becomes evident that mitochondrial proteins in the secretome of male cells are more prone to alteration as a result of hyperoxia. Secreted mitochondrial proteins have been proposed to serve multiple functions, including participating in long‐range metabolic regulation, for example, as a form of quality control, or stimulating the immune system (Sugiura et al. 2014; Suh and Lee 2024; Todkar et al. 2021; Zhang et al. 2010). Mitochondrial dysfunction in male OPCs due to hyperoxia could lead to the secretion of oxidized mitochondrial contents that could act as damage‐associated molecular patterns (DAMPs) and lead to inflammatory cell activation in neighbouring cell types like microglia and astrocytes, and also trigger pro‐inflammatory immune response leading to further damage in the immature male brain. Even though we see that many of the mitochondrial proteins become less abundant in the male secretome post‐hyperoxia, their number is significantly higher than in the female secretome. Additionally, we detected increased carbonylation in CPT2 (carnitine palmitoyl transferase 2) protein enriched from male supernatants post hyperoxia (data not shown) as compared to female samples, thus indicating increased oxidation in the male proteins. However, more studies need to be performed in order to better understand their activities as DAMPs.

Moreover, the presence of FGF‐2 in the female secretome after hyperoxic treatment, together with an upregulation of glutaredoxin‐1 (GLRX‐1), suggests a paracrine mechanism by which female OPCs could enhance the antioxidant capacity and facilitate repair processes in the cells of their local microenvironment extending a possible protective effect to the female brain. FGF‐2 is known to influence the differentiation of OPCs (Zhou and Armstrong 2007), supports angiogenesis, plays an important role in maintaining the integrity of the blood‐brain barrier (Bendfeldt et al. 2007), promotes cell survival and proliferation (Jin‐qiao et al. 2009; Zhu et al. 2018), and can modulate inflammatory responses (Woodbury and Ikezu 2014). FGF‐2 has also been shown to exert neuroprotective effects in multiple models of neonatal hypoxia‐ischemia (Celik, Atici, et al. 2016; Celik, Resitoglu, et al. 2016; Hu et al. 2017; Lin et al. 2017; Ye et al. 2018), and may play a role in other complications of preterm birth, for example, intraventricular haemorrhage (Finkel et al. 2023). Our experimental results clearly demonstrate the beneficial effects of female hyperoxia‐treated OPC supernatants on the survival of differentiated neurons, which can be attributed to the presence of FGF‐2.

GLRX1 plays a direct role in mitigating oxidative stress by reversing protein glutathionylation and maintaining intracellular and extracellular redox homeostasis (Liu et al. 2020). Although we could not link FGF‐2 and GLRX1, together these factors may indicate a robust female‐specific mechanism to counteract hyperoxia‐induced oxidative stress. The selective secretion of these factors by female OPCs also implies inherent sex differences. It would be interesting to further investigate the role of estrogen or other sex‐hormones in upregulating pathways that might enhance GLRX1 and FGF‐2 production. Thus, this clearly demonstrates how hyperoxia can ultimately induce a complex, sex‐specific stress response, integrating antioxidant defence and growth factor signalling to support survival and repair. These findings suggest that targeting FGF‐2 and Glrx pathways could enhance resilience in females, but may also be leveraged to improve outcomes in males. However, in vivo hyperoxia models could be used to further validate these findings.

When discussing sex differences, it is prudent to consider endogenous testosterone levels during early postnatal development, as these may influence OPC responses to hyperoxia. Although direct studies on testosterone–hyperoxia interactions in OPCs are currently lacking, it is well established that male mouse pups undergo a transient surge in testosterone during the first few days after birth (Clarkson et al. 2014). This hormonal preconditioning of the cells prior to isolation may contribute to sex‐specific differences in cellular stress responses, including vulnerability to oxidative injury. Studies in neonatal models have shown that testosterone can exacerbate brain injury by promoting pro‐apoptotic and pro‐inflammatory pathways (Hill and Fitch 2012; Hill et al. 2011; Lingappan et al. 2016). In this context, even though in this study, we did not manipulate hormone levels, it is plausible that higher endogenous testosterone levels in males may sensitize OPCs to oxidative stress, contributing to the differential responses we observed. Future studies manipulating hormonal status will be important to directly test this hypothesis.

These profound sex‐specific differences in the secretome and the associated differential mechanisms of stress response and survival may explain why the neurological outcome in preterm infants is different in males and females. In addition, these findings provide valuable insights into the basic biology of OPCs and how sex can influence cellular function. This could spur further research into the mechanisms driving these differences and how they impact overall brain health and function.

Our study included both male and female samples under each treatment condition, and a formal statistical testing for sex × treatment interactions was conducted according to the recent guidelines on sex‐inclusive research (Garcia‐Sifuentes and Maney 2021; Rich‐Edwards and Maney 2023) that underscores the importance of interaction testing for substantiating sex‐specific effects, (data provided in Table S1). However, we would like to emphasize that, in this paper, due to the limited number of biological replicates (*n* = 3 per group), we present disaggregated group data and individual values to enable transparent interpretation of potential sex‐related trends. These findings are exploratory and warrant validation in future studies with larger sample sizes and sufficient power for interaction testing.

## 4 Experimental Procedures

### 4.1 Animals

Mouse protocols were approved by the Veterinary and Food Control Office, State Department of Agriculture, Food Safety and Fisheries Mecklenburg‐Vorpommern, Germany (Permit Number: ZSF3936/11/17). Animal colonies were housed and maintained following the international FELASA, national GVsolas and local University of Greifswald animal research guidelines. Crl:CD‐1 (ICR) mice were received from Charles River Company and bred for maximal two generations at the facility to get the donor mice. This strain was used to generate oligodendrocyte precursor cells.

### 4.2 Isolation and Culture of Mouse OPCs

Mouse OPCs were isolated from enzymatically dissociated P2‐P4 old CD‐1 (wild type strain) mice brains. For each isolation, tissue from the mid‐brain region comprising the subventricular zone from three male and three female (from the same litter) brains was pooled separately. The sex of the pups was determined visually and by genotyping. All animal usage for cell isolation was performed according to the institutional regulations regarding animal ethics.

Isolated cells were cultured in serum free growth medium supplemented with B‐27 (w/o retinol), endothelial growth factor (EGF, Human Recombinant; Cat. No. GF144; Merck Millipore, Darmstadt, Germany) 0.02 µg/mL and fibroblast growth factor (FGF, Human FGF basic; Cat. No. 100–18B; Peprotech, Thermo Fisher Scientific, Cranbury, NJ, USA) 0.005 µg/mL under 3% O_2_ conditions. After ∼5–6 days, when the neurospheres were approximately 100 µm in diameter, the neurosphere growth medium was gradually replaced with serum‐free B104CM (conditioned media) containing oligosphere medium every alternate day for 2 weeks. After this time period, the cells were trypsinized and used for experiments. A detailed protocol for the isolation and culture of mouse OPCs has been published in Nature Protocol Exchange (Sunny et al. 2015).

To confirm the preservation of OPC identity post‐hyperoxia, the cells were subjected to O4 immunostaining along with O4 and CNPase immunoblotting to quantify protein level changes (Figure S2). Additionally, individual OPC marker protein intensities were separately analysed from the intracellular mass spectrometry data (Sunny et al. 2020), as shown in Figure S2. In addition to these experiments, we also compared our intracellular proteomic dataset (Sunny et al. 2020) with that of Chaerkady et al. (2011), who performed a quantitative temporal proteomic analysis during the differentiation of human embryonic stem cells into OPCs. This comparison revealed a high degree of overlap in OPC‐specific proteins (data not shown), further supporting the identity and purity of the OPCs used in our study.

### 4.3 Cell Lines

B104 Neuroblastoma cell line was purchased from the American Type Culture Collection (ATCC). The cells were used only for preparing conditioned media for OPC culture as described previously (Sunny et al. 2015).

ReNcell VM (mentioned as ReNcell throughout), a human neural progenitor cell line, was acquired from Merck Millipore, Darmstadt, Germany. The cells were used to perform XTT assays and to test the effect of OPC supernatants. ReNcells were cultivated in laminin‐coated culture wares in ReNcell NSC maintenance medium (Cat. No. SCM005, Merck Millipore) supplemented with 20 ng/mL final concentration of EGF and bFGF at 21% O_2_, 5% CO_2_ and 37°C. To generate differentiated neurons, ReNcells were cultured for at least 7 days in NSC maintenance medium supplemented with 20 ng/mL brain‐derived neurotrophic factor (BDNF) and glial cell line‐derived neurotrophic factor (GDNF), B27‐supplement, 1 µM dibutyryl cAMP and 200 µM ascorbic acid (neuronal differentiation media).

### 4.4 Treatment With Different Oxygen Concentrations and Supernatant Collection

The cells from male and female derived OPCs were trypsinized, counted and seeded in equal densities into 6‐well plates for adherent cells (without any coating; Cell+, Sarstedt; Nümbrecht, Germany) containing 1 mL DMEM/F12 + 50.6 mM glucose (666 µL 45% glucose stock solution for 50 mL DMEM/F12) medium per well. Subsequently, the plates were maintained at two different oxygen levels 80% O_2_ (hyperoxia), and 3% O_2_, (normoxia), and incubated with 5% CO_2_ and at 37°C for 24 h. Supernatant was collected from each well without disturbing the cells and was centrifuged at 3000 rpm for 15 min to remove cells and debris. This supernatant was frozen at –80°C and used for the functional assays.

### 4.5 Proteome Analysis by Mass Spectrometry

Concentration of extracellular proteins were determined in a Bradford assay using 200 µL of culture supernatants and DMEM medium as reference. The mean protein concentration was 0.04 ± 0.048 µg/µL.

Intracellular proteins of ReNcells (*n* = 4) were extracted with M‐PER lysis buffer (Thermo Fisher, Waltham, MA, USA) Halt Protease and Phosphatase Inhibitor Cocktail (Thermo Fisher) according to manufacturer's protocol. Protein containing supernatant was collected by centrifugation (16,000 x *g*, 60 min, 4°C) and nucleic acid degraded enzymatically with universal nuclease (0.125 U/µg protein, (Pierce/Thermo, Rockford, IL, USA). Protein concentration was determined by BCA assay (Pierce/Thermo).

Volumes corresponding to 4 µg protein per sample (supernatants: 90–100 µL; intracellular protein: 2.5–5.1 µL) were subjected to the magnetic bead based SP3 protocol (Blankenburg et al. 2019; Sielaff et al. 2017) including reduction and alkylation of proteins, tryptic digestion (16 h, trypsin to protein ratio 1:25) and separation of low molecular weight contaminants from the peptide solution. Peptides were analyzed by LC‐ESI tandem mass spectrometry on QExactive mass spectrometers (Thermo Scientific, Bremen, Germany). Mass spectra recorded in data‐dependent mode for peptides of extracellular proteins (*n* = 3) were analysed by MaxQuant software (v 1.6.2) and Analyst (Genedata, Basel, Switzerland). Mass spectra for peptides of intracellular proteins of ReNcells were recorded in data‐independent mode, which reduced missing values and allowed robust quantification of proteins. Analysis was conducted using Spectronaut v.18 (Biognosys, Zurich, Switzerland). Statistical analysis was carried out on the peptide level after exclusion of peptides with oxidized methionine using the algorithm ROPECA (Suomi and Elo 2017). Only proteins that showed different abundance (adj. *p* value < 0.05 using Benjamini–Hochberg multiple test correction) were used for further considerations. Detailed information on MS data acquisition, database search and data analysis is provided in Table S4. Impact of sex, treatment and the interaction of the terms was tested in a two‐factorial design using the function aov implemented in the R stats package v4.4.0.

### 4.6 FGF‐2 ELISA

Enzyme‐linked immunosorbent assay (ELISA) for FGF‐2 was performed to quantify the amount of FGF‐2 present in the male and female OPC supernatants from different treatment conditions. ELISA was performed in accordance with the protocol stipulated in the Mouse FGF‐2 (bFGF) ELISA Kit (Cat # EMFGF2, Thermo Fisher). For the FGF‐2 ELISA, supernatants from four independent biological replicates were tested.

### 4.7 XTT Cell Viability Assay

The XTT reduction assay is commonly used for the measurement of cell viability. The assay relies on the cleavage of a yellow tetrazolium salt (XTT) to form an orange water‐soluble formazan product by metabolically active, viable cells. Assays were conducted in clear, flat‐bottomed, 96‐well plates with a total volume of 100 µL in each well.

ReNcells were seeded in 96‐well plates with a cell density of 250,000 cells per well. From the next day after seeding, the cells were cultivated in neuronal differentiation media for a period of seven days, during which time they were permitted to differentiate into neurons. Once the neurons were differentiated, they were kept untreated (Ctrl_N), subjected to the OPC supernatant and inhibitors/FGF‐2 for 24 h under normoxic conditions. Comparable hyperoxic controls (Ctrl_H) were generated by maintaining cells at 80%O_2_ for 24 h. After treatment, the viability of the cells was evaluated through the addition of XTT, after which the optical density was measured following a six‐hour incubation period. Absorbance at 555 nm was read with 655 nm as reference wavelength using a Spectra Max Plus plate reader (Molecular Devices, San Jose, CA, USA).

For the XTT assays, supernatants from five different biological replicates were tested.

### 4.8 Quantification and Statistical Analysis

Statistical parameters including the exact value of *n*, the definition of centre, dispersion and precision measures (mean ± SEM) and statistical significance are reported in the figures and figure legends. Data was judged to be statistically significant when *p* < 0.05 calculated by two‐tailed Student's *t* test, if not otherwise indicated.

### 4.9 Data and Software Availability

The mass spectrometry proteomics data have been deposited to the ProteomeXchange Consortium via the PRIDE partner repository (http://www.ebi.ac.uk/pride) with the dataset identifier numbers PXD060307 and PXD060285 for the OPC and ReNcell datasets, respectively.

Metascape, used to analyse part of the proteomic data is a free software available at https://metascape.org (Zhou et al. 2019).

The Ingenuity Pathway Analysis (IPA) software from Qiagen (Hilden, Germany) was utilized for the purpose of functional categorization of proteomic results from ReNcells.

## Author Contributions

**Donna Elizabeth Sunny**: conceptualization, investigation, writing – original draft, methodology, formal analysis, validation, visualization, writing – review and editing, data curation, project administration. **Elke Hammer**: conceptualization, investigation, methodology, writing – review and editing, formal analysis, data curation, supervision. **Stephan Michalik**: writing – review and editing, software, formal analysis. **Uwe Völker**: writing – review and editing, resources, funding acquisition. **Matthias Heckmann**: funding acquisition, writing – review and editing, resources, supervision.

## Conflicts of Interest

The authors declare no conflicts of interest.

## Supporting information




**Supplementary Table S1**: Proteins quantified in the secretome of male and female cells.

**Supplementary Table S2**: Secretome ON/OFF targets.

**Supplementary Table S3**: Results of proteomic analysis of ReNcells after treatment with supernatant of female OPC from either normoxic or hyperoxic condition or FGF‐2 only.

**Supplementary Table S4**: Details of LC‐MS/MS and search parameters (data dependent mode; quantitative data)

**Supplementary Figure SI**: Analysis of apoptosis in OPCs under normoxic and hyperoxic conditions.

**Supplementary Figure SII**: Analysis of OPC markers in male and female OPCs.

Supplementary Information

## Data Availability

The data that support the findings of this study are available in ProteomeXchange Consortium via the PRIDE partner repository at https://www.ebi.ac.uk/pride/, reference number PXD060307 and PXD060285.
