# Supplementary material for: Sex‐Specific Differences in the Secretome of Oligodendrocyte Progenitor Cells Post Hyperoxic Stress
Source: J Extracell Biol. 2025 Sep 23;4(9):e70082. doi: 10.1002/jex2.70082 (PMC12455015; doi:10.1002/jex2.70082)
Supplement: Supplementary file 2 — Supplementary Table S2: Secretome ON/OFF targets. [file JEX2-4-e70082-s006.docx]

**Table S2: Secretome ON/OFF targets.**

Table representing the complete list of proteins (depicted by their gene names) that were identified only in either the normoxic conditions (N) or hyperoxic conditions (H) in male and female samples respectively, along with their reported functions. The column ‘ON/OFF_Male’ represents proteins exclusively detected under normoxic or hyperoxic conditions in samples from male OPCs and similarly ‘ON/OFF_Female’ represents proteins in samples from female OPCs.

The column “Intensity rank” provides information on the intensity rank of proteins only secreted upon hyperoxia (ON/OFF: H).

| Gene name | ON/OFF_Male | Intensity  rank | | | Function | | Reference |  |
| --- | --- | --- | --- | --- | --- | --- | --- | --- |
| ***Prss22*** | N | |  | Tumor cell migration and invasion | | Chen et al., 2014 | | |
| Oacyl | H | | 1 | Acyltransferase activity | | Uniprot.org | | |
| ***Mink1*** | H | | 2 | Regulator of dual leucine zipper kinase (DLK)/c-Jun-N-terminal kinase (JNK) pathway signaling in neurons that drives both neurodegeneration and axon regeneration | | Larhammar et al., 2017 | | |
| Ubtd2 | H | | 3 | Associated with diseases like frontotemporal dementia, Alzheimer’s disease and Amyotrophic Lateral Sclerosis | | Taskesen et al., 2017  Xu et al., 2014 | | |
| Srp14 | H | | 4 | Component of the signal recognition particle (SRP) complex, a ribonucleoprotein complex that mediates the cotranslational targeting of secretory and membrane proteins to the endoplasmic reticulum | | Uniprot.org | | |
| Triobp | H | | 5 | Actin cytoskeleton organization  Regulation of substrate adhesion-dependent cell spreading | | Seipel etal., 2001 | | |
| ***Gnai1*** | H | | 6 | Involved in G protein-coupled receptor signaling pathway  Associated to pediatric encephalopathy | | Solis et al., 2021 | | |
| Mri1 | H | | 7 | Catalyzes the interconversion of methylthioribose-1-phosphate (MTR-1-P) into methylthioribulose-1-phosphate (MTRu-1-P) | | Uniprot.org | | |
| Slc41a3 | H | | 8 | Na+/Mg2+ ion exchanger that acts as a predominant Mg2+ efflux system at the mitochondrial inner membrane | | Uniprot.org | | |
| Creld1 | H | | 9 | Associated with neurodevelopmental phenotypes | | Jeffries et al., 2024 | | |
| ***Pqbp1*** | H | | 10 | Associated with neurodegenerative disorders and brain inflammation | | Jin et al., 2021 | | |
| ***Rassf2*** | H | | 11 | Involved in epidermal growth factor receptor signaling pathway via I-kappaB kinase/NF-kappaB cascade  Positive regulation of apoptotic process and JNK cascade | | Song et al., 2012  Song et al., 2010 | | |
| ***Arih2*** | H | | 12 | Plays an essential role in protein ubiquitylation and degradation | | Martinez-Noel et al., 1999 | | |
| Tmem176b | H | | 13 | Dendritic cell differentiation  Required for the development of cerebellar granule cells | | Condamine et al., 2010  Yukiteru et al., 2006 | | |
|  | **ON/OFF_Female** | |  |  | |  | | |
| ***Prss22*** | N | |  | Tumor cell migration and invasion | | Chen et al., 2014 | | |
| ***Nt5e*** | N | |  | Catalyzes adenosine monophosphate (AMP) breakdown to adenosine  Associated with neuroblastoma outcome | | Jain et al., 2023 | | |
| ***Vgf*** | N | |  | Roles in neurogenesis and neuroplasticity Associated with learning, memory, depression and chronic pain | | Jiang et al., 2019  Lin et al., 2015 | | |
| Znf428 | N | |  | Associated with Amyotrophic Lateral Sclerosis | | Sjoqvist et al., 2023 | | |
| ***Pllp*** | N | |  | Involved in myelination, intracellular transport and lipid raft formation | | Azzaz et al., 2023  Shulgin et al., 2021 | | |
| ***Vps37c*** | H | | 1 | Component of the endosomal sorting complex required for transport  Endosome transport via multivesicular body sorting pathway and protein targeting to membrane  A regulator of vesicular trafficking process  Cellular stress responses when they are associated with ESCRT-I destabilization | | Eastman et al., 2005  Kolmus et al., 2021 | | |
| Osbpl3 | H | | 2 | Associated with Amyotrophic Lateral Sclerosis  Response to glucocorticoids in the brain | | Darbyson et al., 2016  Juszczak et al., 2018 | | |
| Eml4 | H | | 3 | Stabilizes microtubule | | Houtman et al., 2007 | | |
| ***Fgf2*** | H | | 4 | Glial cell differentiation  Response to axonal injury | | Reuss et al., 2003  Jungnickel et al., 2005; Timmer et al., 2007 | | |
| ***H1-1*** | H | | 5 | Bound to vesicles  Play a role in the positive regulation of receptor-mediated endocytosis | | Brix et al., 1998 | | |
| Bpgm | H | | 6 | Plays a major role in regulating hemoglobin oxygen affinity  Known to increase oxyhemoglobin and reduce energy metabolism under stress conditions | | Qiang et al., 2021; Xu et al., 2020 | | |
| ***Arnt2*** | H | | 7 | Transcription factor  Development of the hypothalamo-pituitary axis and postnatal brain growth | | Aitola and Pelto-Huikko, 2003; Hosoya et al., 2001 | | |
| Pcbp3 | H | | 8 | Iron export and heme catabolism | | Ishii et al., 2020  Qiu et al., 2022 | | |
| Itprid2 | H | | 9 | Regulates mitochondrial Ca2+ uptake | | Atakpa-Adaji et al., 2024 | | |
