# Supplementary material for: Sex‐Specific Differences in the Secretome of Oligodendrocyte Progenitor Cells Post Hyperoxic Stress
Source: J Extracell Biol. 2025 Sep 23;4(9):e70082. doi: 10.1002/jex2.70082 (PMC12455015; doi:10.1002/jex2.70082)
Supplement: Supplementary file 4 — Supplementary Table S4: Details of LC‐MS/MS and search parameters (data dependent mode; quantitative data) [file JEX2-4-e70082-s004.docx]

Supplemental Table S4_A) Details of LC-MS/MS parameter (data dependent mode; quantitative data)

| **Data dependent analyses (DDA)** |  |
| --- | --- |
| **reversed phase liquid chromatography** | **Ultimate 3000 RSLC (Thermo Scientific)** |
| trap column | 75 μm inner diameter, packed with 3 μm C18 particles (Acclaim PepMap100, Thermo Scientific) |
| analytical column | 75 μm inner diameter, packed with 2.6 μm C18 particles (Accucore, 25 cm, Thermo Scientific) |
| flow rate | 300 nl/min |
| column oven temperature | 40°C |
| buffer system | binary buffer system consisting of 0.1% acetic acid in HPLC-grade water (buffer A) and 100% ACN in 0.1% acetic acid (buffer B) |
| gradient | gradient of buffer B: 2min 2% to 5 %, 8min 5%, 120min 5% to 25%, 5min 25 to 40%, 2 min 40% to 90%, 5 min 90%, 3 min 90% to 2%, 5 min 2% |
| **Mass spectrometer** | **Q Exactive HF** |
| operation mode | data-dependent |
| electrospray | Nanospray Flex Ion Source |
| **Full MS** |  |
| MS scan resolution | 60,000 |
| AGC target | 3e6 |
| maximum ion injection time for the MS scan | 20 ms |
| scan range | 333 to 1650 *m/z* |
| spectra data type | profile |
| **dd-MS2** |  |
| resolution | 15,000 |
| MS/MS AGC target | 1e5 |
| minimum ACG target | 1e4 |
| intensity threshold | 4e5 |
| maximum ion injection time for the MS/MS scans | 25 ms |
| number of MS/MS scans | Top 15 |
| spectra data type | profile |
| selection for MS/MS | 1 |
| isolation window | 1.4 Da |
| fixed first mass | - |
| dissociation mode | higher energy collisional dissociation (HCD) |
| normalized collision energy | stepped, 27.5 |
| charge exclusion | unassigned,1, 7, 8, >8 |
| dissociation mode | HCD |
| dynamic exclusion | 30 sec |

Supplemental Table S4_B) Details of search parameter for DDA data

| **Parameter** | **Value** |
| --- | --- |
| Name of peaklist-generating software | MaxQuant - Andromeda |
| Name of the search engine & release version | MaxQuant vs. 1.6.2.10 |
| Enzyme specificity considered | Fully tryptic |
| # of missed cleavages permitted | 2 |
| Name of database searched and release version/date | UniProt database release 2020_04 limited to Mus musculus (reviewed only) |
| MS/MS tol. (FTMS) | 20 ppm |
| Include contaminants | False |
| PSM FDR | 0.01 |
| Protein FDR | 0.01 |
| Min. peptide Length | 6 |
| Min. score for modified peptides | 40 |
| Modifications included in protein quantification | Oxidation (M);Acetyl (Protein N-term) |
| Max mods in site table | 3 |
| Match between runs | True |
| Matching time window [min] | 0.7 |
| Max. peptide mass [Da] | 4600 |
| Threshold score for accepting protein identification | 1 significant unique peptides |
| Label min. ratio count | 1 |
| Rest of the parameters were set to default. |  |
|  |  |

Supplemental Table S4_C) Details of LC-MS/MS parameter (data independent mode; quantitative data)

| **Data independent analyses (DIA)** |  |
| --- | --- |
| **reversed phase liquid chromatography** | **Ultimate 3000 RSLC (Thermo Scientific)** |
| Trap column | 75 μm inner diameter, packed with 3 μm C18 particles (Acclaim PepMap100, Thermo Scientific) |
| Analytical column | 75 μm inner diameter, packed with 2.6 μm C18 particles (Accucore, 25 cm, Thermo Scientific) |
| Flow rate | 300 nl/min |
| column oven temperature | 40°C |
| buffer system | binary buffer system consisting of 0.1% acetic acid in HPLC-grade water (buffer A) and 100% ACN in 0.1% acetic acid (buffer B) |
| gradient | gradient of buffer B: 2min 2% to 5 %, 8min 5% to 7%, 60min 7% to 25%, 5min 25 to 40%, 2 min 40% to 90%, 6 min 90%, 2 min 90% to 2%, 10 min 2% |
| **Mass spectrometer** | Exploris 480 mass spectrometer (Thermo Scientific) |
| operation mode | data-independent |
| electrospray | Nanospray Flex Ion Source |
| **Full MS** |  |
| MS scan resolution | 120,000 |
| Normalized AGC target | 300 % |
| maximum ion injection time mode | custom |
| Scan range | 350 to 1200 *m/z* |
| Spectra data type | profile |
| **dd-MS2** |  |
| Resolution | 35,000 |
| MS/MS AGC target | 3.00E+06 |
| maximum ion injection time for the MS/MS scans | auto |
| Spectra data type | profile |
| selection for MS/MS | 1 |
| isolation window | 65 windows *m/z* 13, overlap *m/z* 2 |
| Fixed first mass | 200 |
| dissociation mode | higher energy collisional dissociation (HCD) |
| normalized collision energy | fixed, 30% |
| dissociation mode | HCD |

Supplemental Table S4_D) Spectronaut parameters for peptide/Protein identification and intensity extraction from DIA data

| Spectronaut 18.7 |  |
| --- | --- |
| **Pulsar Search\Peptides** |  |
| Toggle N-terminal M: | True |
| Min Peptide Length: | 7 |
| Max Peptide Length: | 52 |
| Missed Cleavages: | 2 |
| Digest Type: | Specific |
| Enzymes / Cleavage Rules: | Trypsin/P |
| **DIA Analysis\Data Extraction** |  |
| MS1 Mass Tolerance Strategy: | Dynamic |
| Correction Factor: | 1 |
| MS2 Mass Tolerance Strategy: | Dynamic |
| Correction Factor: | 1 |
| Intensity Extraction MS1: | Maximum Intensity |
| Intensity Extraction MS2: | Maximum Intensity |
| **DIA Analysis\XIC Extraction** |  |
| XIC IM Extraction Window: | Dynamic |
| Correction Factor: | 1 |
| XIC RT Extraction Window: | Dynamic |
| Correction Factor: | 1 |
| **Pulsar Search\Modifications** |  |
| Max Variable Modifications: | 5 |
| **Database** |  |
| Original File: | Uniprot_reviewed_N20433_02_2024 (*Homo_sapiens*) |
| **Select Modifications:** |  |
| Fixed Modifications:: | Carbamidomethyl (C) |
| Variable Modifications: : | Acetyl (Protein N-term), Oxidation (M) |
| **DIA Analysis\Calibration** |  |
| MS1 Mass Tolerance Strategy: | System Default |
| MS2 Mass Tolerance Strategy: | System Default |
| **DIA Analysis\Identification** |  |
| Machine Learning: | Per Run |
| Protein Qvalue Cutoff (Experiment): | 0.01 |
| Exclude Single Hit Proteins: | False |
| PTM localization | True |
| Probability cut-off | 0.75 |
| Pvalue Estimator: | Kernel Density Estimator |
| Precursor Qvalue Cutoff: | 0.001 |
| Single Hit Definition: | By Stripped Sequence |
| **DIA Analysis\Quantification** |  |
| Amino acids | False |
| Interference Correction: | True |
| MS1 Min: | 2 |
| MS2 Min: | 3 |
| **Fragment ions:** |  |
| Best N fragments per peptide | True |
| Min | 6 |
| Max | 10 |
| Ion Charge | False |
| Ion Type | False |
| Protein LFQ Method: | Automatic |
| Major (Protein) Grouping: | by Protein Group Id |
| Minor (Peptide) Grouping: | by Stripped Sequence |
| Modifications | None |
| Minor Group Top N: | False |
| Minor Group Quantity: | Sum precursor quantity |
| Major Group Top N: | True |
| Min: | 2 |
| Max: | 3 |
| Major Group Quantity: | Mean peptide quantity |
| Quantity MS-Level: | MS2 |
| Quantity Type: | Area |
| Proteotypicity Filter: | None |
| Data Filtering: | Qvalue |
| Imputing Strategy: | Use background signal |
| Cross Run Normalization: | True (local normalization) |
| **DIA Analysis\Workflow** |  |
| MS2 DeMultiplexing: | False |
| Run Limit for directDIA Library: | -1 |
| Method Evaluation: | False |
| Profiling Strategy: | iRT Profiling |
| Profiling Row Selection: | Minimum Qvalue Row Selection |
| Qvalue Threshold: | 0.001 |
| Profiling Target Selection: | Profile only non-identfied Precursor |
| Identification Criterion: | Qvalue |
| Threshold: | 0.001 |
| Carry-over exact Peak Boundaries: | False |
| Unify Peptide Peaks Strategy: | Select corresponding Peak |
| **DIA Analysis\Post Analysis** |  |
| Calculate Sample Correlation Matrix: | True |
| Calculate Explained TIC: | None |
| Differential Abundance Grouping: | Major Group (Quantification Settings) |
| Smallest Quantitative Unit: | Precursor Ion (Quantification Settings) |
| Group-Wise Testing Correction: | False |
| Run Clustering: | True |
| Distance Metric: | Manhattan Distance |
| Linkage Strategy: | Ward's Method |
| Z-score transformation: | False |
| Order Runs by Clustering: | True |
| **DIA Analysis\Protein Inference** |  |
| Protein Inference Workflow: | Automatic |
| Inference Algorithm: | IDPicker |
