# Supplementary material for: Sex‐Specific Differences in the Secretome of Oligodendrocyte Progenitor Cells Post Hyperoxic Stress
Source: J Extracell Biol. 2025 Sep 23;4(9):e70082. doi: 10.1002/jex2.70082 (PMC12455015; doi:10.1002/jex2.70082)
Supplement: Supplementary file 5 — Supplementary Figure SI: Analysis of apoptosis in OPCs under normoxic and hyperoxic conditions. [file JEX2-4-e70082-s002.docx]

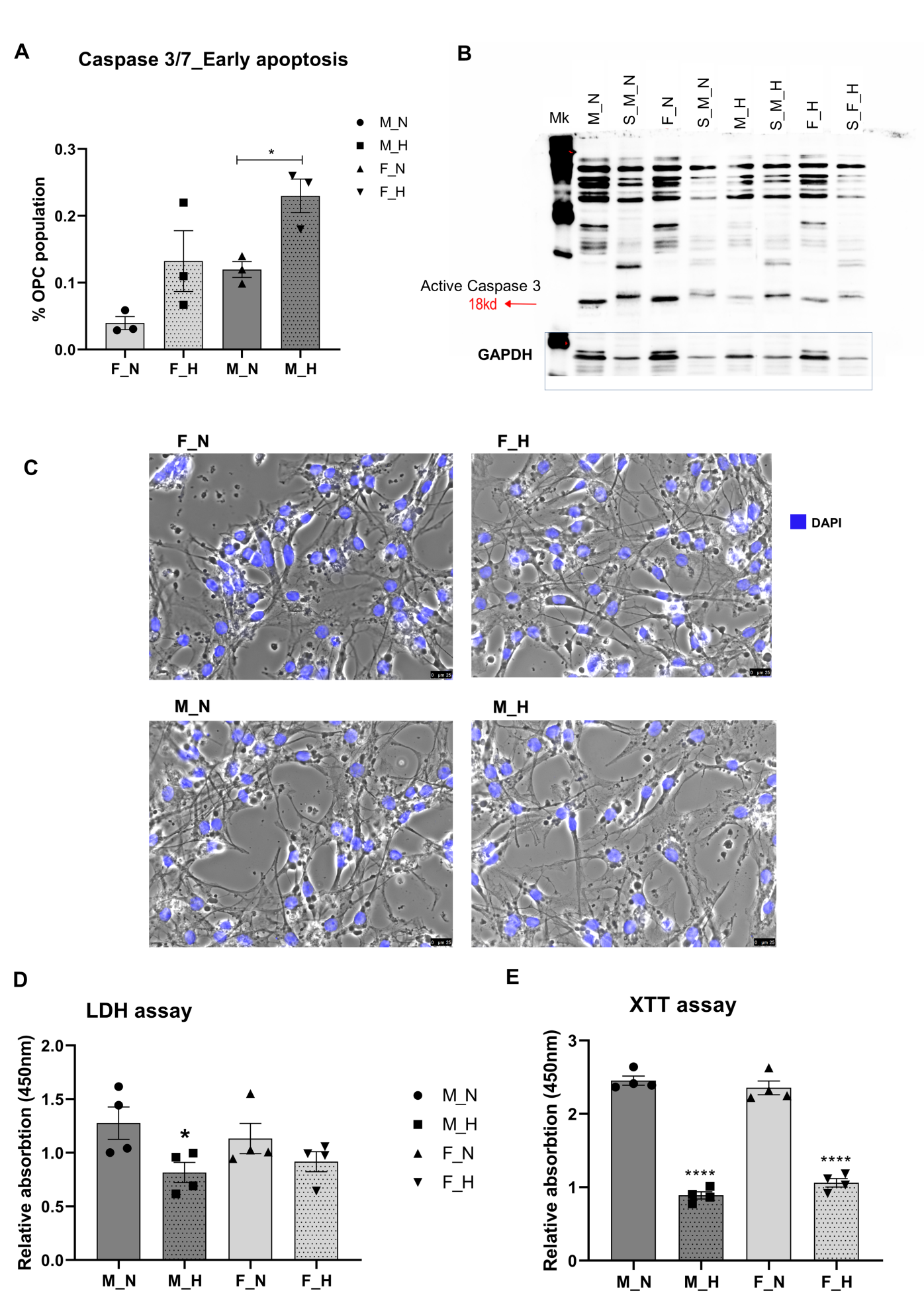


Figure I: A) Graphical representation of flow cytometry results of caspase 3/7 apoptosis assay performed on OPCs post 24h 80% O_2_ treatment. F_N represents female normoxia, F_H - female hyperoxia, M_N – male normoxia and M_H – male hyperoxia.

B) Western blot membrane image probed with active caspase 3 antibody showing expression of active caspase 3 at 18kd. In the lane annotation, the first lane marked ‘M’ stands for protein marker, in the following sample annotations,‘C’ stands for Control-untreated, ‘M’ for male, ‘N’ for normoxia, ‘H’ for hyperoxia and ‘S’ for staurosporin treated positive control.

C) Representative phase contrast images of male and female OPCs treated or non-treated with hyperoxia for 24h, stained with DAPI (blue). Scale bar represents 25uM.

D) LDH release assay results showing significantly decreased LDH in male culture supernatant post 24h hyperoxia treatment.

E) Graph showing the effect of 24h hyperoxia treatment on the viability of OPCs as determined by XTT assay. Increased specific absorbance values indicate higher metabolic activity and hence cell viability.

Data are representative of at least 3 independent bio-replicates.

Bars and error represent mean ± SEM of replicate measurements. *p < 0.05, ****p < 0.0001 (Student’s t test).
