# Supplementary material for: Sex‐Specific Differences in the Secretome of Oligodendrocyte Progenitor Cells Post Hyperoxic Stress
Source: J Extracell Biol. 2025 Sep 23;4(9):e70082. doi: 10.1002/jex2.70082 (PMC12455015; doi:10.1002/jex2.70082)
Supplement: Supplementary file 6 — Supplementary Figure SII: Analysis of OPC markers in male and female OPCs. [file JEX2-4-e70082-s007.docx]

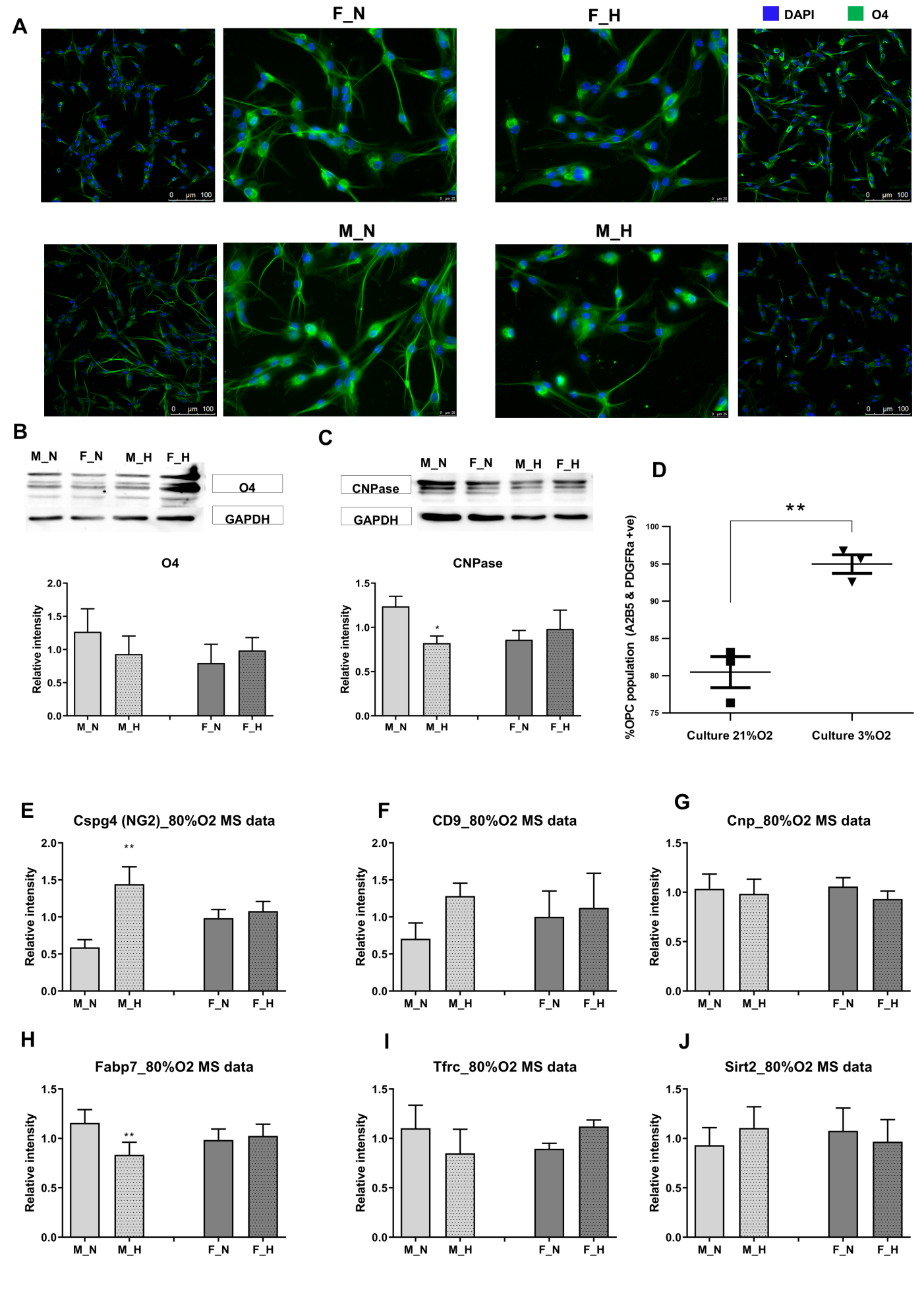


Figure II A) Representative images of mouse male and female OPCs stained for the OPC marker O4 after treatment under normal oxygen conditions and post 24 h 80% O_2_. Scale bar of the extreme left and right images represents 75 μm and 4 images in the center represents 25um.

B) Western blot analysis of male and female OPCs with anti-O4 and C) anti-CNPase antibodies under normal (3% O_2_) conditions and post 24 h 80% O_2_ treatment, showing a significant decrease in expression of only CNPase in the male OPCs. Whereas in female OPCs, both O4 and CNPase protein expression remained almost unchanged, or only very slightly increased.

D) FACS data showing the difference between the percentages of A2B5 and PDGFRα positive OPCs at 3% and 21%O_2_ culture conditions. FACS staining was performed using Anti-A2B5-APC antibody (Miltenyi Biotec) and PE anti- mouse CD140a antibody (Biolegend).

E-J) Graphs showing the relative intensities of OPC markers, E) Cspg4 (NG2), F) CD9, G) Cnp, H) Fabp7, I) Tfrc and J) Sirt2 as plotted by using the individual intensity data for each bio-replicate sample from the mass spectrometric analysis (Sunny *et al*., 2020).

Data are representative of 3 independent bio-replicates.

Bars and error represent mean ± SEM of replicate measurements. *p < 0.05, **p < 0.01 (Student’s t test). M_H- male hyperoxia (80 % O_2_), M_N- male normoxia (3% O_2_), F_H- female hyperoxia, F_N- female normoxia.
