# Supplementary material for: Sex‐Specific Differences in the Secretome of Oligodendrocyte Progenitor Cells Post Hyperoxic Stress
Source: J Extracell Biol. 2025 Sep 23;4(9):e70082. doi: 10.1002/jex2.70082 (PMC12455015; doi:10.1002/jex2.70082)
Supplement: Supplementary file 7 — Supplementary Information [file JEX2-4-e70082-s003.docx]

**Immunofluorescence**

For immunofluorescence staining (IF), cells were fixed in 4% paraformaldehyde, rinsed twice in 1x PBS and incubated at room temperature in a blocking solution (3% normal goat serum in 1x PBS + 0.3% Triton x100), followed by incubation with primary antibodies at 4 °C overnight. Samples were then washed 3 times with phosphate buffered saline-Tween 20 (PBST) and incubated with fluorescence conjugated secondary Alexa antibodies (Life Technologies) at room temperature for 2 h. Slides were mounted with Vectashield Mounting Medium with Dapi (Vector Laboratories, UK) and imaged on Leica SP5 microscope (Leica, Germany).

**Immunoblot analysis**

Protein extracts from whole cell lysates containing ∼ 40 μg protein were loaded in each lane of a Mini-Gel module for electrophoresis (BioRad, Munich, Germany). Protein was transferred onto nitrocellulose membrane (Amersham Protran 0.45 μm NC Western Blotting Membrane, GE Healthcare, USA), blocked with 1x Blocking buffer (Pierce™ Protein-Free (TBS) Blocking Buffer, Thermo Fisher) at RT for 1 h, and incubated in primary antibody at 4 °C overnight. GAPDH (Rabbit Anti-GAPDH (D16H11) mAb, Cell Signaling Technology) was used as the loading control at 1:1000 dilution. Blots were incubated with secondary antibody at 1:10000 dilution in blocking buffer for 1 h at RT. Protein bands were visualized with SuperSignal™ West Femto Maximum Sensitivity Chemiluminescence Substrate (Thermo Scientific). Densitometric intensities were calculated using the ImageLab software (BioRad).

**Fluorescence-activated cell sorting (FACS) and flow cytometry**

To assess marker expression, OPCs were harvested and dissociated using TrypLE (Thermo Fisher). The pellet was resuspended in ice cold FACS buffer (PBS + 0.5% BSA + 0.1% Sodium azide) and filtered through a 40 µm cell strainer to obtain a single-cell suspension. Cells were incubated with fluorophore-conjugated antibodies against OPC surface markers [anti-PDGFRα-PE, anti-A2B5-APC] for 60 minutes at 4 °C in the dark. Following incubation, cells were washed and resuspended in FACS buffer. Viability was assessed using propidium iodide in separate tubes. Data were acquired on a BD FACSCanto II flow cytometer and analyzed using FlowJo software (BD Biosciences). Appropriate fluorescence minus one (FMO) and isotype controls were included to define gating strategies.

Primary antibodies used for the above-mentioned methods are as listed below:

Anti-A2B5-APC antibody (Miltenyi Biotec)

PE anti- mouse CD140a antibody (Biolegend)

Anti-mouse Oligodendrocyte Marker O4 antibody (R&D systems)

Anti-CNPase antibody (Abcam)

FGF-2 Rabbit Polyclonal antibody (Proteintech)

**LDH Cytotoxicity Assay**

Cytotoxicity was assessed by measuring lactate dehydrogenase (LDH) release into the culture medium using the LDH-Cytotoxicity Assay Kit (Abcam, ab65393), according to the manufacturer’s protocol. After 24 hours of exposure to hyperoxia or normoxia, culture supernatants from male and female OPCs were collected and transferred to a 96-well plate. The LDH reaction mixture was added to each well and incubated at room temperature for 30 minutes in the dark. Absorbance was measured at 450 nm using a microplate reader. Background signals were corrected using culture medium alone, and differential LDH release between the different treatment groups was represented using relative absorbance values.

**Caspase-3/7 Activity Assay by Flow Cytometry**

Early apoptosis was assessed using the CellEvent™ Caspase-3/7 Green Flow Cytometry Assay Kit (Life Technologies, Thermo Fisher Scientific), following the manufacturer's instructions. After 24 hours of hyperoxia or normoxia exposure, male and female OPCs were harvested, washed with PBS, and resuspended in the supplied assay buffer. Cells were incubated with the CellEvent™ Caspase-3/7 Green Detection Reagent (final concentration: 5 μM) for 30 minutes at 37 °C in the dark. To exclude dead cells, SYTOX™ AADvanced™ Dead Cell Stain was added during the final 5 minutes of incubation. Samples were analyzed using a flow cytometer, and data were processed using FlowJo software. Caspase-3/7-positive, SYTOX-negative cells were classified as early apoptotic.
